# Molecular Microbial Community Analysis as an Analysis Tool for Optimal Biogas Production

**DOI:** 10.3390/microorganisms9061162

**Published:** 2021-05-28

**Authors:** Seyedbehnam Hashemi, Sayed Ebrahim Hashemi, Kristian M. Lien, Jacob J. Lamb

**Affiliations:** 1 Department of Energy and Process Engineering & Enersense, Norwegian University of Science and Technology (NTNU), 7034 Trondheim, Norway; seyedbehnam.hashemi@ntnu.no (S.H.); ebrahim.hashemi@ntnu.no (S.E.H.); kristian.m.lien@ntnu.no (K.M.L.); 2 Department of Electronic Systems, Norwegian University of Science and Technology (NTNU), 7034 Trondheim, Norway

**Keywords:** microbial diversity, next generation sequencing, proteomics, metabolomics, community diversity, anaerobic digetion, biogas

## Abstract

The microbial diversity in anaerobic digestion (AD) is important because it affects process robustness. High-throughput sequencing offers high-resolution data regarding the microbial diversity and robustness of biological systems including AD; however, to understand the dynamics of microbial processes, knowing the microbial diversity is not adequate alone. Advanced meta-omic techniques have been established to determine the activity and interactions among organisms in biological processes like AD. Results of these methods can be used to identify biomarkers for AD states. This can aid a better understanding of system dynamics and be applied to producing comprehensive models for AD. The paper provides valuable knowledge regarding the possibility of integration of molecular methods in AD. Although meta-genomic methods are not suitable for on-line use due to long operating time and high costs, they provide extensive insight into the microbial phylogeny in AD. Meta-proteomics can also be explored in the demonstration projects for failure prediction. However, for these methods to be fully realised in AD, a biomarker database needs to be developed.

## 1. Introduction

**Figure 1:**
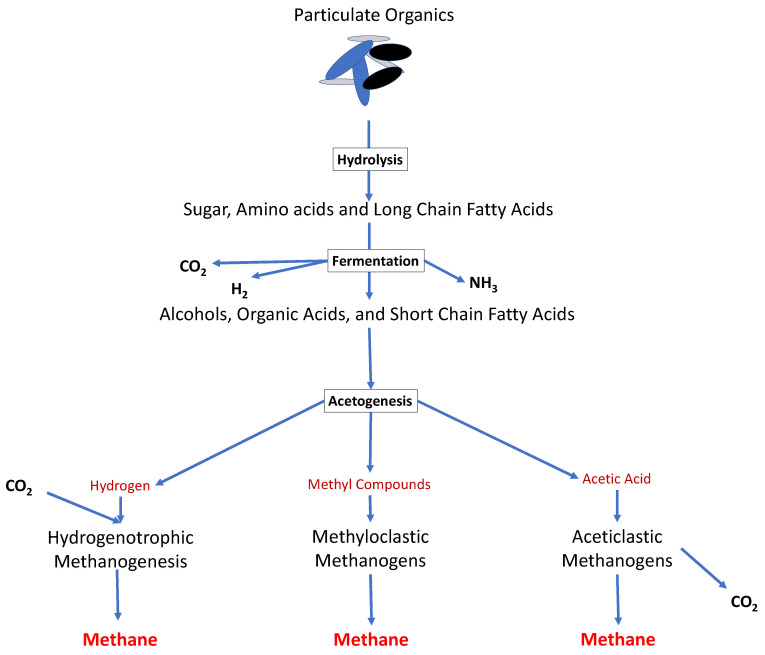
The overall metabolic pathways of anaerobic digestion. Polymeric organic materials go through the four stages of anaerobic digestion (hydrolysis, acidogenesis, acetogenesis and methanogenesis), in order to produce raw biogas.

Microorganisms are abundant organisms in the environment that play essential roles in the sustainability of all life on the Earth [1]. Anaerobic digestion (AD) is an engineered process for biological waste management through the conversion of the organic feedstocks by microorganisms to produce biogas (i.e., a mixture of mainly methane and carbon dioxide) [2]. The AD process is a metabolic reaction consisting of four main steps in series (i.e., hydrolysis, fermentation or acidogenesis, acetogenesis and methanogenesis), where several types of anaerobic bacteria and archaea interact together to produce biogas (Figure 1) [3,4,5,6]. Moreover, the AD process is strictly dependent on the activity of the microorganisms, and its efficiency is affected by critical interactions between the microorganisms (i.e., known as syntrophic activities (which can be defined as close cooperation between at least two organisms based on the transfer of metabolic products from one to another)) within the digester [2,7].

Although the online monitoring of conventional operation parameters (e.g., pH, temperature, volatile fatty acids, biogas composition and alkalinity) reflect the current situation in terms of monitoring AD, these do not provide enough data to understand the microbial community composition, dynamics and function, limiting the predictability of the process direction [8,9]. Additional information such as electron transfer mechanisms [10,11], the level of functional equivalence in the microbial networks and the metabolic capacity of newly identified microorganisms are required to develop and optimize AD [12,13]. Meta-omic techniques and gene amplicon sequencing methods can fill this gap in the understanding of AD and have been developed in order to link the function and activity of the microbial community [14,15].

This manuscript starts by giving an overview of the anaerobic digestion process and continues by presenting microbial biology and molecular techniques relevant for use in AD. The authors emphasize the importance of the meta-omics techniques in terms of understanding the complete picture of microbial community diversity and interactions in AD systems. Finally, the review highlights the gaps between different disciplines and how to bridge these in order to achieve a comprehensive modelling approach in AD. This paper gives a broad summary of gaps in the field by suggesting currently employed solutions that will help to develop an advanced mathematical model for AD. It also aids in developing stable and predictable AD while helping to identify new pathways for biogas production.

## 2. Anaerobic Digestion (AD)

During hydrolysis, complex polymers (including carbohydrates, proteins and lipids), are converted to the simpler monomers such as monosaccharides (C5 and C6 sugars), amino acids and long-chain fatty acids (LCFAs) [5]. Different types of facultative and obligate anaerobes produce extracellular enzymes to accomplish the hydrolysis process [4,16]. Acidogenesis is carried out with acidogenic bacteria. In this step, approximately 70% of the products from hydrolysis of carbohydrates are converted to hydrogen, carbon dioxide and acetate that can be directly used by methanogenic archaea [17]; however, for proteins and fats, this conversion ratio can be different. For instance, proteins first convert to amino acids and then to final products. For lipids, the hydrolysis splits them into LCFAs and glycerol, where the LCFAs are decomposed through β-oxidation [18]. The remaining hydrolysis products are converted into intermittent components including short-chain fatty acids (e.g., propionate, butyrate and alcohols), which require further degradation [6,19].

The intermediate products of acidogenesis are further fermented by acetogenic organisms to acetate, methyl compounds, carbon dioxide and hydrogen [20]. Methanogenesis is mainly carried out by a group of Archaea organisms known as methanogens, which can be categorized in three main groups [21]. The main active methanogens are aceticlastic methanogens that produce methane from acetates through the aceticlastic pathway, hydrogenotrophic methanogens that produce methane from CO_2_ reduction by hydrogen or formate, and methyloclastics methanogens that are capable of consuming methyl compound (e.g., methanol, methylamines and methyl sulphide) to produce methane [4,22,23].

Hydrogenotrophic methanogens play a crucial role in the stability and robustness of the AD by maintaining low hydrogen partial pressure in the system. Most of the methane in AD is produced by aceticlastic methanogens (i.e., approximately two-thirds), with minimum methane generation from methyloclastic methanogens [21]; however, in high ammonia content (>3 g/L NH3-N) [24], the methane production pathway from acetate will be reduced. In this condition, the hydrogenotrophic methanogens provide the dominant methane production pathway [25,26,27]. The typical growth rate of different functional groups varies by several factors including the type of substrate, operational condition, microbial diversity and the type of reactor [28,29]. It is generally accepted that the hydrolysis of complex substrates such as lignocellulosic materials are the rate-limiting step in AD [30]. However, additional factors may result in low growth rates for proton-reducing syntrophic bacteria and methanogenic microorganisms in AD [31], especially in continuous feeding reactors where some of the microbes will leave the reactors through the outlet [32]. For example, methanogens are very sensitive to environmental disturbances such as pH, accumulation of fatty acids and ammonia concentration, while the growth rate of proton exchange bacteria is extremely dependent on the partial pressure of hydrogen in the system [32,33].

The acetogenic activity is thermodynamically affected in the high partial pressure of hydrogen; moreover, the conversion reaction of butyrate and propionate to acetate and hydrogen will only proceed in a low concentration of hydrogen in the system [34,35]. This can be avoided through balancing hydrogen production from acetogens and hydrogen consumption by methanogens [4,36]. Under certain conditions (e.g., high temperature and high ammonium concentration), where the acetolactic methanogenesis pathway is inhibited, an alternative pathway will be opened to convert acetate to hydrogen and CO_2_. This pathway links the syntrophic acetate oxidation by acetate-oxidizing bacteria to hydrogenotrophic methanogenesis as shown in Table 1.

Even though the acetate oxidation pathway through acetate-oxidizing bacteria is not the main biological pathway in most biogas production plants, it can become the dominant methane generating pathway in high operating temperatures and high ammonia concentrations [18]. In AD, syntrophic species produce H_2_ and acetate through degrading organic materials (e.g., fatty acids, alcohols and aromatic compounds) and in endergonic reactions (i.e., an additional driving force is needed to perform these reactions) [37]. From the acetate oxidation reaction (Table 1) it can be concluded that the only factor that causes the reaction toward hydrogen production is the low concentration of hydrogen on the right side of the reaction. The feasibility of such reactions depends greatly on hydrogen consumption by methanogenesis to maintain low hydrogen partial pressure in the system. In addition, the syntrophic species (e.g., acetate oxidizing-bacteria) are also dependent on physical attachment to the electron accepting cells (e.g., hydrogenotrophic methanogens) to ease immediate interspecies electron transfer. AD is based on such H_2_ production/consumption partnerships [38].

Even though the general scheme of AD is well understood, the biology behind AD is not entirely established. Optimized AD cannot be achieved by simply identifying the microorganisms, but also requires determination of activity and interaction between different microorganisms [12].

## 3. Microbial Diversity

In microbiology, a microbial culture can be developed in order to determine types of microbe or for testing the absence of specific organisms. A microbial culture is used to regenerate and grow microorganisms on special growth media [39,40]. Methods that are based on growing and selecting microbes through conventional cultivation media (often on conventional cultivation media in Petri dishes) in a specific ecosystem are known as culture-dependent techniques [41,42]. Only 1% of the known microorganisms can be cultivated in this way due to several factors including lack of specific nutrients, oxygen level, temperature, pH, biological interactions and missing growth factors (e.g., an important element that can be produced by other microbes in the original culture) [39].

The culture-dependent techniques (e.g., DNA-DNA hybridization) are suitable to identify the main population in a specific metabolic process. Since the culture-dependent techniques do not consider the environmental factors affecting the AD process, the application of these techniques alone does not give a complete picture of the microbial ecology and physiology of the system [12,43,44].

In the past, synthetic media was the main option for cultivation. With the advent of polymerase chain reaction (PCR) the analysis of microorganism without cultivation became possible. Several methods were developed and coupled with PCR for investigating complex cultures, and as a result the culture-independent technique was coined. This term includes the modes that are not based on cultivation [41]. Culture-independent methods (e.g., cloning of 16S rRNA and denaturing gradient gel electrophoresis) rely on molecular methods to study microbes within their original environments. These approaches, together with supplementary techniques including imaging, chemical analysis and isotope labelling, can give a better insight into the microbial community and its dynamics. Such culture-independent techniques have exposed previously uncharacterized microbial diversity in AD [12].

Microbial diversity is the range of microorganisms and their relative abundancy in a specific community [45]. Microbial diversity is important because it has an effect on process robustness [46]. Each species in the biological system has its own weaknesses. If the environmental situation pushes these species toward their inhibition, then, in a diverse culture, other species can maintain their activity through other metabolic pathways to compensate for the lack of specific activities in the system. As a result, a metabolically diverse system is a stable and robust biological culture for various environmental conditions [22].

The microbial diversity can give precise information regarding the biological diversity in three main levels (i.e., genetic variation within a species, distribution and number of different species and the community diversity or ecology). However, the classification of unknown bacteria can be the main challenge in the determination of microbial diversity [47]. The biodiversity can be estimated by measuring the divergence in molecular characters (i.e., by nucleic acid homology). The stability of the system is related to the stability of the community, and stress within the system can lead to an unstable system and variation in the species diversity [48]. Therefore, diversity analysis is of interest as it provides a better understanding of [47]:

- the genetics of the organisms and their distribution in the community
- the functional role of their diversity
- the types of species
- the specific amount of each species within the system

Abiotic factors (e.g., temperature, pH, oxygen, nutrients and toxic materials), together with morphological characterization of cells (i.e., cell shape, cell wall structure and flagella per individual cell) are not enough to establish a detailed classification of the microbe. Therefore, biotic factors at the molecular level (e.g., the DNA sequence) must be analyzed to obtain stronger classification of the microbial system [49].

Recently developed methods in molecular and chemical ecology have introduced promising options in order to study the microbial diversity [50,51]. These methods can be used to investigate the microbial diversity and community structure. They are classified into molecular biology and biochemical techniques that are comprehensively reviewed by Fakruddin et al. [47] (Table 2).

Polymerase chain reaction (PCR) amplification is a standard molecular biology method allowing the amplification of specific DNA sequences that can be used to determine the microbial community composition. The small subunit rRNA genes (i.e., 16S rRNA) are regularly employed to study the biodiversity and community composition for many microbial systems [12]. Traditional molecular fingerprint methods or first-generation sequencing techniques have been effectively used for the assessment of the microbial community in anaerobic digesters; however, these methods are time-consuming and give a low resolution of the community [53].

**Figure 2:**
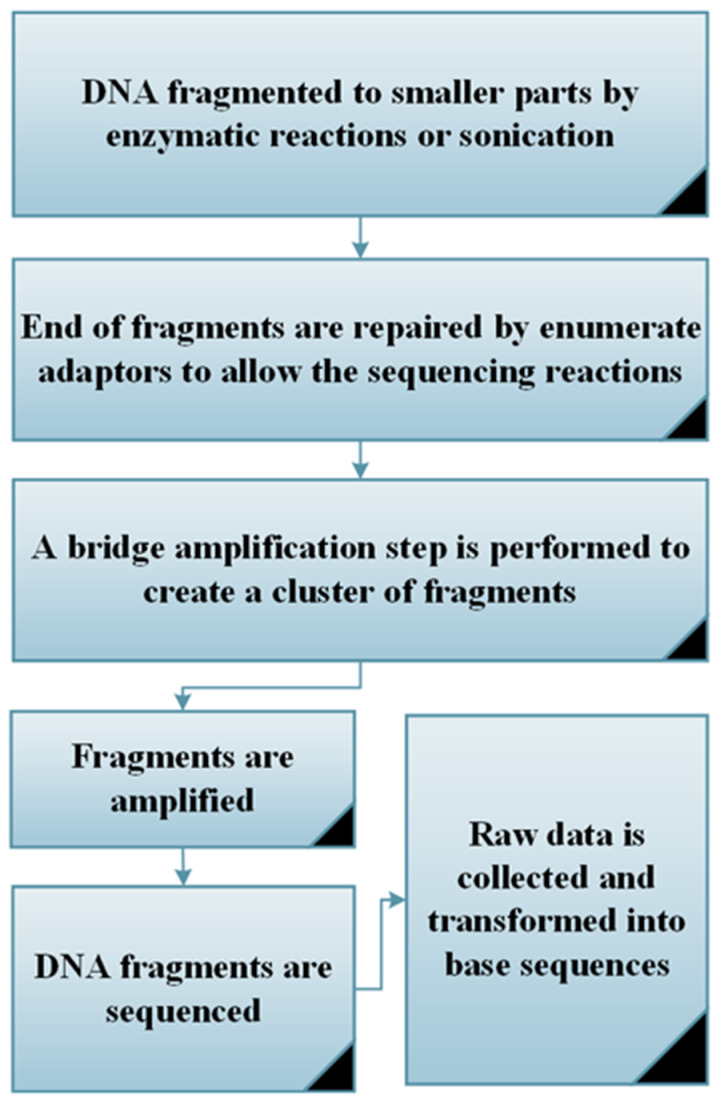
The 6 main steps for conducting a next-generation sequencing (NGS) approach.

Unlike traditional sequencing technologies, recently developed sequencing technologies, known as high-throughput techniques or next-generation sequencing (NGS), are capable of sequencing multiple DNA molecules simultaneously with low cost, short processing times and high resolution [54]. These features lead to the generation of large data sets that can enhance the statistical correlation analysis [12]. Six main steps are adapted for NGS techniques as shown in Figure 2 [55].

Illumina and Roche 454 are two main high-throughput platforms for sequencing 16S rRNA that have been used for AD culture analysis. In fact, the correlation between the community composition and the operational conditions (e.g., feed type, temperature, ammonia concentration, pH and organic loading rate) can be investigated by the data collected from high-throughput sequencing techniques [6,56]. Additionally, the combination of long-term operation monitoring, and microbial diversity may reflect significant information regarding community function. Werner et al. [57] showed that to maintain syntrophic populations, resilience is more important than the dynamic competition. Moreover, they demonstrated a strong relationship between methanogenic activities and substrate removal efficiency [57]. Overall, the improved resolution of high-throughput sequencing methods has led to the discovery of thousands of operational taxonomic units (OTU) in full-scale AD (an OTU can refer to a group of individual microbes with some similarities including unknown organisms with DNA sequence similarity), while earlier this number was as low as 69 OTUs (i.e., examined with clone libraries) [57,58]. Moreover, this high resolution can aid in the identification of low abundant populations and their contribution to biogas production [12].

Among the high-throughput techniques, pyrosequencing (employed by the 454 Roche platform) is widely used to assess community composition in AD. For the first time, Ronaghi et al. [59] introduced pyrosequencing based on employing pyrophosphate (PPi) and produced the sequencing through a synthesis reaction; however, this pyrosequencing approach has some limitations, and modified techniques were required. These limitations and modifications in the PCR have been well-reviewed by Ari and Arikan 2016 [60]. The 454 Roche system can only generate a low amount of short reads (i.e., it produces a 400 megabase (unit of DNA fragments length) sequence in each run with an average read length of 400 basepairs) [12].

Reversible dye terminator (RDT) methods were developed for wider sequencing ranges [60]. RDTs are categorized into two main classes: blocked and unblocked (bRDT and ubRDT, respectively) [60,61]. bRDT has shown better performance in the termination process (mainly used for second-generation sequencing), and ubRDTs are more efficient in sequence elongation results [62]. The Illumina platform is a second-generation sequencing platform based on bRDT that utilizes 3′-O-azidomethyl for DNA sequencing [60]. The Illumina MiSeq platform can generate 2 × 300 basepair paired end reads and 4 terabases of sequence per run, which is significantly larger than the 454 Roche platform [12].

Since the quality of the community composition analysis is important, several studies have attempted to evaluate the possible errors associated with applying NGS techniques [63,64]. For example, pyrosequencing provides an overestimation of rare phylotypes (i.e., a group of small rRNAs that have a specific level of similarity in gene markers) due to artificial amplification. Even though the sequencing results from Illumina do not contain these limitations, it has some systematic calling biases (i.e., tiles of sequencing plates tend to produce reads of different quality) [60,65]. The complexity of downstream analysis, for example by de novo genome assembly, will be lowered by reducing these errors from the sequencing process. There are methods under development for the estimation and correction of these shortfalls [66]. Many correction algorithms including accurate correction of error (ACE) [67], Bayes Hammer [68], bloom filter-based error correction solution (BLESS) [69] and error correction (EC) [70] have already been developed. The error correction tools identify and correct the sequences through replacing uncovered k-mers (i.e., from the k-mer coverage spectrum of the input data) with k-mers that have a higher coverage [66]. It has been shown that the error correction algorithms reduce the sequencing errors without introducing new sources of error [71]. Short-read assemblers first generate a de Bruijn graph containing all k-mers (sub-sequences of length k contained in biological sequences) of the input reads and their overlaps [66]. Consequently, the presence of error sequences introduces an additional analysis task.

## 4. Determination of Metabolic Functionality by Meta-Omic Techniques

### 4.1. Metagenomics

NGS-based metagenomics is a rapidly growing research field in different biological systems including the human body, animals, soil, ocean and anaerobic environments, which aids the understanding of the diversity and functional complexity [72,73]. In AD, a metagenomic method can provide insight into the progress of a digester. An example is the ability to follow the AD process from the initial step, through an acidic condition (i.e., where volatile fatty acids (VFAs) are accumulated), and back to its normal operation condition [73,74]. The main objective of metagenomic methods, especially in a less complex environment, is to rebuild large fragments of genomes (or complete genomes) from species present in the microbial community [75,76].

In more complex environments such as AD, gene-centric metagenomics have shown better performance by providing an overview of gene frequency [77,78]. Metagenomic techniques have revealed a high number of gene reads, of which most of them are not yet identified, and consequentially, the functional information from these reads is limited. Despite this, in AD, metagenomics have provided insight into the evolutionary relationships among different species and the metabolic functionality of the microbial community [12].

Feedstock type, substrate pre-treatment and the operational conditions can significantly affect the function and diversity of the microbial community [46,79]. Combined metagenomics and the AD performance data is a technique through which the functional redundancy can be estimated. In addition, it is possible to achieve a stable operational condition by maintaining the level of metabolic diversity [80]. Higher resolution and longer read length of future amplicon sequencing methods, together with improved algorithms and genome binning methods, can introduce future advances in metagenomics [81,82].

In the near future, rebuilding near-complete genomes by metagenomics combined with other meta-omic methods (e.g., meta-transcriptomes and meta-proteomes) not only assists generating a strong genomic database for AD, but can also provide information with respect to the interaction between various functional groups [12].

### 4.2. Meta-Transcriptome

Meta-transcriptomics is the study of the function and activity of the complete set of transcripts (mRNA sequence) from the culture sample. Besides measuring the in-situ gene expression, it gives an insight into the activity of microbes (i.e., the genes that have increased or reduced expression in a specific microbial environment) [83]. Meta-transcriptomic approaches reduce the complexity of metagenomics by focusing on the species that are suggested to be metabolically active [1,83]. In order to calculate the gene expression, the reads from these approaches (i.e., generally 20 million reads are sufficient), need to be mapped against reference genomes (e.g., metagenome reads from the same environment). Therefore, it is a faster, cheaper and a more reliable technique that, unlike traditional methods such as measuring by microarrays, can also detect novel genes [83].

High-throughput transcriptomics have increased the measuring of gene expression profiles and have enabled the identification of unknown sequence transcripts. Despite this, meta-transcriptomics introduce practical challenges and limitations including low recovery of high-quality RNA and the short half-life of mRNA, difficulties enriching mRNA, and bias related to cDNA synthesis and amplification [84,85]. Some of these problems can be addressed through developing protocols, enhancing sequencing platforms and improving sampling and storage methods [1,12]. For instance, to avoid deterioration of RNA quality, the best method is to immediately extract RNA samples then store at −80 °C or snap freeze in liquid nitrogen and store in −80 °C [86]; however, the longer the storage, the more deterioration in the quality of RNA observed. The other possible way to reduce loss of RNA is avoiding the use of RNALater (i.e., a liquid tissue storage reagent that stabilizes cellular RNA in an unfrozen condition) to maintain the integrity of the samples as it lyses some cells and can interfere with the RNA extraction kit (e.g., Ambion RiboPure Bacteria Kit/ LifeTeh Trizol Plus). Using kits for sample preparation is another important factor to recover more mRNA and removing abundant ribosomal RNA species. Traditionally, the recovery of the mRNA was approximately 25%, while the recently developed kits can recover mRNA to around 70% [87,88].

### 4.3. Meta-Proteome

**Figure 3:**
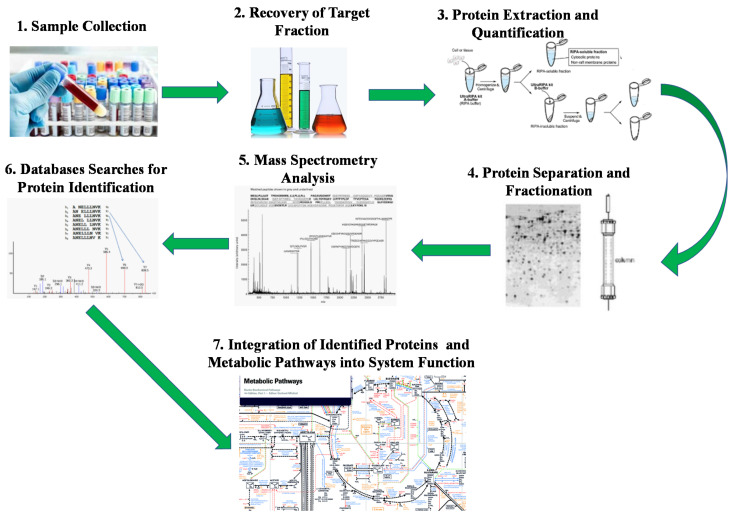
Typical workflow for (meta) proteomics analysis [based on [91]]. Initially, the samples are collected from the biological community then the microbial fraction or extra cellular components are separated from the sample. After protein extraction, gel-based or gel-free methods can be used to separate proteins that are then analyzed through mass spectrometry (e.g., peptide sequence or peptide mass fingerprint). After determining the peptide amino acids, tandem mass spectra of peptides can match database-derived protein sequences and finally the proteins can be linked to different metabolic pathways.

Wilmes and Bond [89] have defined meta-proteomics as the identification of all the expressed proteins at a given time within an ecosystem under a specific condition (e.g., soil, sediments, marine and freshwater, anaerobic environments, human body and animal guts). Metaproteomic approaches are based on six main steps (Figure 3). The metaproteomic analysis methods include all data extracted from metagenomics, diversity and functional diversity information and identified biological processes in order to define the metabolic activity and biological pathways of the microorganisms along with their cooperation and competition [90]. Siggins et al. [91] gives an extensive overview of this process, and the sampling and analysis method for AD samples have been described by Heyer et al. [92].

The main superiority of meta-proteomics in AD is the possibility of linking the function of the proteins with a certain taxonomy and correlating their presence with metabolic activity [93]. Abram et al. [94] conducted a meta-proteomics approach that revealed the enzymes involved in different pathways including methanogenesis from CO_2_/acetate, glycolysis and pentose phosphate pathways. Heyer et al. [95] showed that the microbial community is shaped by syntrophic interactions, competition and phage-induced interactions causing a slower biogas production process due to cell lysis. They discovered that meta-proteomics can be used to investigate and identify the metabolic activities within a microbial community and exposed the syntrophic and competitive interactions among different organisms. The main limitations and challenges associated with the application of meta-proteomics approaches in AD are [93]:

- contamination by the products of biomass degradation
- sample complexity
- redundant protein identifications
- lack of detailed databases

Meta-proteomics has been used to create a prototype database for biogas plants [93]. An optimized workflow with low pre-fractionation of samples and high coverage of proteins has been established through employing sensitive Obritrap mass spectrometers and searching spectra against biogas plants metagenomics by comprehensive bioinformatic platforms. Accordingly, the results of these studies showed that the metaproteins or taxonomy could be identified as biomarkers for biogas plants [92,93,95]. Even though some meta-proteomics methods (e.g., gel electrophoresis and cluster analysis) are quite fast and can be used in full-scale AD plants to predict the AD failure, the lack of a comprehensive database for AD biomarkers can reduce the application of such technology [96]. More experimental data have been provided in the section “Possible Applications of Molecular Techniques in AD”.

### 4.4. Meta-Metabolome

Metabolites are the intermediates or end products of metabolism. Meta-metabolome analysis techniques characterize and evaluate the metabolites including metabolic intermediates, hormones and low-weight molecules within an organism (i.e., necessary molecules for maintenance, growth and normal function of microorganisms) [97]. Therefore, the meta-metabolome can provide a snapshot of cellular processes that are taking place or have recently occurred [98]. The main challenge associated with metabolomics is that the detection of metabolites is dependent on knowledge of the biological pathways [98]. Although several methods have been developed to analyze metabolites, the liquid-liquid extraction and analyses (i.e., via gas chromatography-mass spectrometry (GC-MS) or liquid chromatography-mass spectrometry (LC-MS)), coupled with chemometrics, have shown promising results [99].

## 5. Determining Specific Function of Specific Genes and Protein

Meta-omic approaches help to link the known species in the microbial community to relevant metabolism within AD. The understanding of the microbial community can further be improved by gaining insight into substrate consumption via specific species and visualizing the spatial organization of the community.

### 5.1. Stable Isotope Probing (SIP)

DNA stable-isotope probing was originally developed to assess the metabolic function of microorganisms in the environment [100]. This can help to identify the active consumers of substrates within the environment [101]. DNA-SIP (stable isotope probing) has been effectively used to identify functionally active microorganisms in different environments such as soil, cave water, coal mine, freshwater, marine and anaerobic environments [101,102,103]. This approach relies on the incorporation of stable-isotope components (e.g., _13_N or _15_N) into microbial DNA during growth on labelled substrates [100,102]. Combining SIP and metagenomic sequencing approaches is a strong approach to discover novel active organisms within a microbial community [101,103].

### 5.2. Fluorescence In Situ Hybridisation (FISH)

Fluorescence in situ hybridization (FISH) is an extensively researched method to detect specific organisms within a biological sample. The FISH technique relies on employing fluorescently labelled oligonucleotide probes bound to rRNA [104,105]. For the first time in an anaerobic environment, Raskin et al. [106,107] used FISH to specify methanogens within a sample. Although FISH is a suitable tool to identify, quantify and analyze the dynamics of specific organisms in AD, it should be noted that the physiological properties of FISH vary by the operational condition [104,108]. The dependency of FISH on ribosomes reduces the reliability of the general probs. This leads the method to employ specific probes in the sub-groups, requiring information that is unknown [109]. The main advantages and disadvantages of FISH methods for use in different anaerobic environments have been comprehensively summarized by Sanz et al. [110].

### 5.3. Microautoradiography

Microautoradiography (MAR) has been used to assess microbial growth and substrate competition within a culture [111]. The MAR technique employs radioactive isotopes to investigate substrate uptake by specific organisms [12,112]. The main advantage of MAR is its high sensitivity and short incubation time; however, its dependency on radioactive isotopes with relatively long half-lives limits its application. Despite this, Carman [113] investigated the consumption of radioactively labelled substrates by a sedimentary microorganism using MAR. The results showed that the [_3_H]acetate and [_3_H]thymidine were consumed by heterotrophic bacteria and the [_14_C]bicarbonate was taken up by microalgae. A combined MAR-FISH technique has also been applied to investigate the metabolically active microbial cells. Ito et al. [44,114] used MAR-FISH with [U-_14_C]glucose to identify the major acetate consumers in AD (i.e., unknown bacteria and filamentous archaea cells), and calculated a substrate degradation rate (i.e., glucose, propionate and acetate) to define the rate-limiting step. Furthermore, to classify undefined acetate consuming bacteria, the MAR-FISH was coupled with SIP and 16S rRNA sequencing [44,114]. The results revealed that the acetate utilizing bacteria belong to the *Synergistes* group four are significantly capable of acetate uptake and had a higher maximum uptake rate than *Methanothrix*. Therefore, these bacteria are more competitive for acetate consumption over *Methanothrix* at high acetate concentrations (2.5 to 10 mM).

### 5.4. Secondary Ion Mass Spectroscopy (SIMS)

Single-cell approaches of stable isotope-labelled cultures use secondary ion mass spectrometry (SIMS) to overcome the limitations of MAR or FISH-MAR [115]. Although these methods are relatively expensive, they can provide promising information on the function and interaction of microbes in their natural habitat. SIMS is a suitable tool to visualize the distribution of stable or radioactive isotopes in microbial cells. SIMS can be used to investigate the cellular function of multicellular organisms (e.g., filamentous cyanobacteria), and is suitable to study the adaptation of microorganisms in their natural environment [116,117].

**Figure 4:**
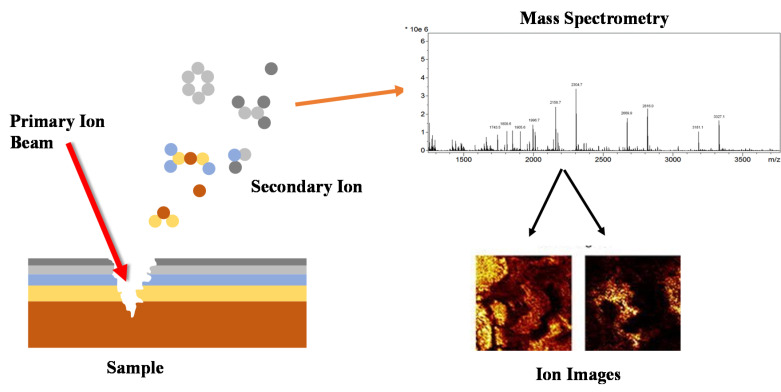
Schematic illustration of a dynamic secondary ion mass spectrometer (SIMS). An ion gun supplies a high energy ion beam (several keV) on the surface of a target biofilm. This results in ionization of the surface and sputters some of the atoms. These atoms will be collected by ion lenses and separated according to their atomic mass. Finally, the ions are exposed to an electron multiplayer or a Faraday cup.

SIMS is a sensitive mass spectrometric technique that measures elemental, isotopic or molecular composition of a solid surface. Initially, SIMS employs an ion gun to produce an ionic beam and generates secondary particles including atoms and molecules (depending on SIMS mode) from the surface of the organism in a high vacuum [115]. Figure 4 gives a schematic representation of the SIMS method. Modern SIMS-based instruments (i.e., NanoSIMS) are capable of measuring up to seven elements or isotopes simultaneously.

A combination of FISH and NanoSIMS can be used to enhance the resolution, aiding the calculation of the substrate uptake rate [118]. Ho et al. [119] analyzed the substrate accumulation and the effect of the hydraulic retention time when the operational temperature varies from 35 °C to 55 °C by NanoSIMS using 13C-labeled acetate. They further analyzed the microbial community through 16S rRNA-based FISH to identify the key species and the active methane production pathways under shorter hydraulic retention times. Werner et al. [120] conducted a long-term experiment (3-year observation of lab-scale AD) based on the FISH-NanoSIMS technique to study the effects of high ammonia loading and identification of alternative pathways for aceticlastic methanogenesis. They reported that the syntrophic acetate oxidation bacteria have controlled the distribution of bacterial phylotypes. It was also observed that a partial shift from aceticlastic to hydrogenotrophic methanogens occurred due to higher ammonia loadings.

The combination of available techniques described above can provide a culture-independent method to identify specific functions and pathways. Further development of NanoSIMS may aid the detection of enzymes, proteins and mRNA transcripts for expression of specific genes and release of key enzymes [12,121].

## 6. Possible Applications of Molecular Techniques in AD

The possible applications of molecular techniques in biogas plants are reviewed and their potential contribution in the improvement of the overall performance of biogas plants is addressed. The major engineering applications of molecular techniques related to meta-omics methods is investigated separately in the following.

### 6.1. 16s rRNA Gene Sequencing

Real-time tracking of 16s rRNA through currently developed facilities can aid in the achievement of a stable AD process [122]. The short analysis time or real-time PCR, digital droplet PCR (i.e., a typical run for real time PCR and digital PCR takes approximately 2 h) and Illumina techniques may lead to their near online applications in AD to continuous track of the total and active bacteria community that can be compared with a reference database [123]. In general, the variations by reason of the operational condition, substrate characterization and presence of inhibitors in community composition can be detected by knowing the composition of a stable community as a reference unit [124]. By linking the 16s rRNA analysis results to these variations, a local database for the biogas plant can be developed to predict the process failures [8]. Nordgård et al. [123] employed Illumina sequencing of 16S rRNA amplicons to specify the effects of the alteration of ammonia concentrations on bacteria and archaea community compositions. They categorized Syntrophomonadaceae as the most abundant OTUs that were positively correlated to methane production in high and low ammonium concentration conditions [123]. They also showed that after long operation periods and culture adaptation (for 200 days), the Aceticlastic *Methanothrix* actively contributed in methane production in low ammonia concentration, while in high ammonia concentration, the *Methanosarcina* played an important role for methane production [123].

16S rRNA-based assessment of AD can be linked to the function of closely related species in the microbial community; however, it should be considered that closely related species can act differently in a microbial community [12,15,125]. Metabolism of an anaerobic culture can also be inferred by relating it to the dynamics and composition of the microbial diversity. In AD, where the competing species and syntrophic groups are responsible for fulfilling the biological pathways, this correlation becomes even more important. Due to complex microbial interactions within AD, the correlation analysis needs to be done carefully. In this way, the hypotheses regarding the correlation between community composition and the metabolic functionality can be developed and further evaluated using supplementary techniques [126].

Ziganshin et al. [6] employed a PCR-based 16s rRNA technique to investigate the effects of using various types of agricultural substrate on microbial community composition in AD. Clostridia and Bacteroidetes were the main bacteria taxa, while Methanomicrobiales and Methanosarcinales were the dominant methanogenic archaea. The results from correlation analysis showed that the community composition was mainly affected by inhibition concentration and the temperature shift from 38 to 55 °C [6]. Conventional substrates such as maize silage combined with cattle manure had a similar and stable community, while chicken manure and Jatropha press cake digested in a less diverse community mainly due to presence of inhibitors. Digestion of chicken manure depended on syntrophic acetate oxidation as the aceticlastic methanogenesis pathway was inhibited, whereas fiber-degrading organisms (*Actinomyces* and *Fibrobacter*) are required for Jatropha press cake digestion [6].

Beside all the positive sides of this method, the 16S rRNA sequencing method has several limitations. These limitations include slow evolution rate, comprising multiple non-identical 16SrRNA genes in addition to difficulties associated with the occurrence of homologous recombination and horizontal gene transfer [127].

### 6.2. Metagenomics

Metagenomics can identify and characterize all the genes and consequently all the organisms present in the AD microbial community. Moreover, metagenomics has revealed dramatic differences between community compositions when the community structure seems similar. Metagenomics can also be employed to expose the potential of each microbial group and their contribution to the biogas production process. For instance, Jaenicke et al. [75] employed gene-centric metagenomics to characterize a full-scale AD process for the first time. In addition to the identification of new taxa, the results revealed that the Clostridia class has a direct effect on reductive CoA or Wood-Ljungdahl pathway (a set of biochemical reactions used by many bacteria and archaea including sulfate-reducing bacteria, acetogens, and methanogens where CO_2_ is reduced to CO and formic acid or formyl group in the presence of two enzymes (CO dehydrogenase and acetyl-CoA synthase). Then the formyl group is reduced to the methyl group. Bacteria and archaea use a reverse reaction for acetate degradation to the methyl group and CO then methylotrophic methanogens reduce the methyl group to methane through CO oxidation to CO_2_ and H2 [128]) (i.e., a syntrophic association between Clostridia and hydrogenotrophic methanogenesis was discovered) [129]. Wirth et al. [130] investigated the microbial community and metabolic functionality of a full-scale AD process fed by maize silage and pig manure. They found that the driving force for optimal biogas production relied on a balance between electron consumers and producers. Hydrogen is an electron transfer mediator meaning that the proton reducers pick up a proton and put on an electron and then the resulting hydrogen is transferred. The proton receiver then grabs the hydrogen and strips off the electron and releases the proton again. There are other electron transfer mediators in the system such as formate or even intraspecies electron transfer methods [10,11,131]. Therefore, the balance in production and consumption of these electron transfer mediators has a significant effect on the stability of AD. Lie et al. [74] added granular activated carbon (GAC) into upflow anaerobic sludge bed (UASB) reactors (i.e., fed by municipal wastewater) in order to assess the increase in the electron transfer among syntrophic bacteria and methanogens. The study indicated that the GAC can enhance methane production through enhanced electron transfer. Moreover, it revealed that *Geobacter* and *Methanosarcina* take the main role in this activity. The abundance of gene coding also concluded that the GAC can inhibit the nitrate- and sulfate-reducing bacteria resulting in an increase of the concentration of methanogenic Archaea.

Metagenomic approaches are appropriate strategies for different objectives such as identifying and isolating key players of an anaerobic culture [132,133]. Metagenomics can also be used in bioprocessing in order to identify effective enzymes aiding degradation of resistant substrates such as cellulose and lignin [134]. In a full-scale plant, detailed information regarding the community composition and the possible potential of the different microbial classes can be extracted by metagenomics. By linking this information to the different operational conditions, a valuable database is provided to understand the strengths and weaknesses of the microbial community against different substrates, presence of inhibitors, and characteristic variations for rapid adaption to the new condition [72,135]. Despite this, the identification of these variations through metagenomics is normally slow compared to the sludge residence times in the anaerobic reactors. This means, in AD, only a limited online application of the currently developed metagenomics can be expected.

### 6.3. Meta-Transcriptome

Although a rich genome/metagenome reference database for AD has not yet been established, meta-transcriptomics has the potential to expose highly expressed pathways for organic conversion in AD. For example, by using meta-transcriptomics, the function of low abundance organisms and their effect on process stability can be determined and documented. Zakrzewski et al. [136] conducted a meta-transcriptomic study on a full-scale AD process. The study revealed the expression of a transcript profile encoding enzymes for each step of AD. A large portion of the reads in this study could not be assigned to the well-known sequences suggesting a need for an extended genome and metagenome reference database. Jia et al. [137] employed genome-centric meta-transcriptomes to identify the active population in the microbial community and rebuilt their metabolic network in an anaerobic culture fed by a cellulose-rich substrate. This study showed that at 35 °C, *Clostridium cellulolyticum*-related bacteria were the dominant organisms performing cellulose hydrolysis, while *Ruminococcus*-related bacteria were the dominant organisms carrying out acidogenesis and acetogenesis.

Meta-transcriptomics allows the determination of the functionality of multi-task organisms (e.g., *Clostridia*, which is capable of different activities ranging from hydrolysis to acetate oxidation), as well as estimation of the dominant route for methane production in AD [6,12,14,15]. Ardèvol et al. [138] carried out a metagenomic and meta-transcriptomic analysis of AD of the microalgae *Spirulina* identifying that the substrate was hydrolyzed by Bacteroidetes (i.e., ML635J-40 aquatic group), and the hydrogenotrophic pathway was the main methane-producing pathway for this type of substrate.

The meta-transcriptomics have been recently applied in the study of electron transfer between syntrophic acetogens and methanogens [126,139]. The results from these studies suggest the possibility of direct electron transfer between *Geobacter* and *Methanothrix* populations meaning that the *Methanothrix* can function as a hydrogenotrophic organism in a direct interspecies electron transfer (DIET) technique even though they have previously been thought to be acetate consumers for methane production. In these approaches, developed biofilms on conductive materials (i.e., granular activated carbon or microbial aggregates derived from an up-flow anaerobic digester) have been analyzed to gain a better understanding of electron transfer concepts in AD [139,140,141]. Shrestha et al. [141] employed meta-transcriptomic techniques to investigate the DIET and H_2_ interspecies transfer (HIT). The gene transcript abundance was assessed in two cultures including *Geobacter metallireducens* and *Pelobacter carbinolicus* that perform DIET and HIT, respectively, and *Geobacter sulfurreducens* as the main electron acceptor. The transcript abundance showed high presence of the pilus-associated cytochrome OmcS (i.e., essential genes for DIET), in the coculture with *G. metallireducens* and a bit higher *G. sulfurreducens* uptake hydrogenase genes in the *P. carbinolicus* culture. These results suggest the ability of the meta-transcriptomics as a route to investigate unique gene expression patterns for DIET and HIT.

By controlling the rate of the conversion of DNA to RNA of gene expression, organisms can be adapted to sudden changes in the surroundings [84]. This refers to the potential application of meta-transcriptomics to explain contradictory results observed in 16S-based studies [142]. For example, meta-transcriptomics make the changes in genetic profiles tangible due to the increase or decrease of system stability caused by the substrate loading rate. Transcriptional level control through meta-transcriptomics in anaerobic cultures can be used to track the changes in metabolic profiles, shifts in the balance of the major functional groups and responses to inhibitory factors (e.g., pH, VFA, partial H_2_ pressure and ammonia concentration) [12,142,143]. In this way, by identifying different pathways and linking them to operational conditions, it can be possible to push the AD towards a stable operation condition with high efficiency [144].

### 6.4. Meta-Proteomics

Through meta-proteomics measures, the level of cellular localization and regulation occurring at the protein/enzyme level can be observed, which can be significantly different from those observed at the genome and transcript level [145,146]. Hanreich et al. [147] conducted a combined metagenomics and meta-proteomics analysis to investigate the hydrolyses of plant carbohydrates in AD. The metagenomic results of this study revealed a minor population of methanogens, while the results of meta-proteomics showed high expression of the methanogens meaning that the methanogens were highly active even with minor abundance [147].

Meta-proteomics, like meta-transcriptomics, can aid process efficiency and stability by providing information about the enzymes and their effects on the AD process and identifying biomarkers as predictive indicators of process failure [147]. Heyer et al. [96] applied meta-proteomic analysis to full-scale agricultural biogas plants. They extracted proteins through sodium dodecyl sulfate polyacrylamidegel electrophoresis (SDS–PAGE) and then used mass spectrometry (MS) to identify the proteins. The study revealed that the protein profiles were specific for each biogas plant and were stable for a long period until disruption (e.g., process acidification) occurred within the system [93,95,96]. This study was able to predict the acidification of AD due to disappearance of major bands in the SDS-PAGE. Moreover, the methyl CoM reductase of *Methanosarcinales* was identified as a reliable biomarker of future process failure. Heyer et al. [96] concluded that the MS methods are expensive and time-consuming methods (significant time is required for sample preparation and analysis), while the approaches based on gel electrophoresis and cluster analysis can be conducted in shorter time frames, with regular application methodologies using capillary electrophoresis allowing significantly faster analysis. This could allow meta-proteomics to be employed in order to monitor full-scale anaerobic digesters.

### 6.5. Meta-Metabolome

The meta-metabolome can also provide more detailed information regarding key metabolic pathways. This is mainly due to higher changes in metabolites related to changes at the transcriptome or proteome level [148,149]. A suitable metabolic biomarker for biodegradation is one that is produced along with biodegradation and is released from the microbial cell, is specific for the bioprocess being monitored and is chemically and biologically stable [98].

Scaglia et al. [150] measured tens of thousands of meta-metabolomes by LC-MS in an AD process fed by manure; however, the application of metabolomics to the whole AD process is limited due to the large range of metabolites and lack of proper knowledge about all biological pathways. Therefore, it is important to use different levels of metabolomics combined with performance data for screening of the under-lying mechanisms [12]. For example, acetyl CoA contributes to syntrophic acetate oxidation as well as conversion of CO_2_ to acetate. Determining only this enzyme (i.e., through meta-proteomics) cannot reflect an exact picture of the process pathway; therefore, other biomarkers must be used to give a better overview of the biological pathways [151].

## 7. Application of Microbial Diversity Analysis in AD Models

By mathematical modeling of AD, a biological process can be designed with the aim of the utilization of a specific type of substrate. Modeling can also help to predict the performance of the biological process and biogas obtained, process conditions and variations over time [152]. Mathematical models of AD have been developed based on the scientific findings regarding the well-known pathways, the interactions among microorganisms, the presence or absence of specific organisms and the final products of each step. These models can be used to evaluate several parallel phenomena leading to in depth insight into the AD process [152].

### 7.1. Metabolic Models of AD

Metabolic modeling is a tool to characterize and predict the function of the microbial community based on knowledge and assumptions of cellular metabolisms that are gained from genome-scale assessments or observations at process level [153,154,155,156]. Consequently, the metabolic modeling for AD is categorized as cellular-level modeling (CLM) or biochemical process modeling (BPM).

The meta-omic approaches can be employed in CLM to demonstrate the cellular fluxes at a specific metabolic state by using assumed or genome-level determination of metabolic limitations of cells in the given condition; however, the pathway or transition to a new metabolic state is not mathematically clear. In this way, the potential impacts of genetic or chemical limits on a metabolic branch can be investigated [154,157]. Through identification of new microbial groups and their function in the AD of different substrates, several models for metabolic reactions have been continuously developed [158]. BPM is focused on the process function of various biological processes including AD. BPM considers the functional groups (e.g., acidogens, acetogens and methanogens) [153]. The well-known examples of BPM in wastewater and AD are the activated sludge model (ASM) [159] and the anaerobic digestion model (ADM) [18].

In AD models, the main research gaps appear in the acidogenesis, acetogenesis and methanogenesis steps. These gaps include regulation mechanisms in the fermentation of sugar; reduction and oxidation of amino acids (Stickland fermentation); electron transfer modes; interspecies signaling and anabolic dependency; competition and acetogen/ methanogen type; and thermodynamic limitations of methanogenic archaea [160]. A better understanding of the functional clades can improve our understanding of the AD process and help bridge these research gaps.

### 7.2. Development of Metabolic Models and Main Gaps in the Field

The basic mathematical models of AD include different microbial functional groups that make a combination of different metabolic pathways possible. These consist of fermentation, acetogenesis, and hydrogenotrophic and aceticlastic methanogenesis [153]. These fundamental models from the 1980s have been developed and have been employed for different AD systems. Finally, in 2002, all the models amalgamated as the IWA AD model No. 1 (ADM 1) [18]. ADM 1 is a four-stage process model including different subgroups that represents the microbiological species utilizing different products in each step of AD; however, the basic ADM 1 has been modified to meet new pathways (e.g., acetate oxidation pathways) and other by product gases such as H_2_S [161]. ADM 1 is established to describe different phenomenon such as pH regulation and acid/base interrelation [18]. This BPM can also present the growth, inhibition and decay of the functional groups including methanogenesis archaea and functional clades of bacteria mediation acidogenesis and acetogenesis [155,162]. Even though these BPMs have been validated for different AD, questions regarding community composition and functions within the major clades have not been answered yet.

The BPM approaches generally consider a mixed microbial culture and introduce proton and electron regulations of NADH for thermodynamic restrictions. Over 30 years ago, Mosey employed this method to regulate the propionate versus acetate production via NADH [163]. The process was controlled through different factors including pH and hydrogen concentration [163]. This method is further developed to predict the fermentation products due to introducing multiple competing regulation mechanisms (e.g., electron bifurcation and acetogenesis). In addition to NADH, the electron carriers in this system extended to FADH and Ferredoxin (Fd). NADH and FADH count as intracellular electron carriers, while Fd is considered as the electron bifurcation element that is capable of direct hydrogen production [155,164].

Unlike sugar fermentation models, models for amino acid fermentation are not well developed since sugar fermentation is a more attractive field for industrial biotechnology compared to amino acids and hydrolyzed proteins. Ramsay and Pullammanappallil (2001) reviewed BPM of pared Stickland fermentation (i.e., Stickland reactions involve the oxidation of amino acids to a carboxylic acid with one carbon shorter than original amino acid), in which one amino acid acts as the electron acceptor and the other is the electron donor, and some specific amino acids act as electron acceptors, donors or both [165,166,167]. In ADM 1, the protein fermentation stoichiometry is stable, but because of the uncoupled oxidation of amino acids it significantly deviates from Stickland fermentation [18].

Modeling of methane production (the final electron sink) from acetate require obligate syntropy between bacteria and archaea [168]. Alternatively, other organisms such as sulfate, nitrate and metal reducing organisms can replace the methane producing archaea to generate different final electron sinks such as hydrogen sulfide [168]. In order to achieve efficient syntrophic oxidation reduction among bacteria and archaea, not only should the amount of electron carriers (e.g., hydrogen and formate) maintain at very low levels, but the presence of specific organics and their close physical coupling are also important [169]. Nonetheless, several research questions still exist including the competing level of other electron acceptors such as sulfur-reducing bacteria, non-substrate coupling, role of different acetate consumers and multiple factors in selecting methanogenic partner (e.g., substrate capacity and affinity, cellular geometry and spatial orientation). A multi-species CLM approach investigates the two latter factors [170]. A complex metabolic interaction network of hydrogen, formate and acetate transfer particularly for complex substrates was observed in this study. The complexity of this network was increased by interdependencies such as amino acid synthesis. The usage of antimicrobials by specific species increased the stability of the interspecies network leading to stable collaboration among community members [170].

The main acetate consuming organics in the AD are subjected to competition [168]. These functional clades are the obligate aceticlastic *Methanothrix*, hydrogen or acetate consuming *Methanosarcina* or other syntrophic acetate oxidizing organisms (e.g., acetate oxidizing bacteria). The *Methanothrix* are dominant at low ammonia (moderate temperature), and *Methanosarcina* and acetate oxidation take over in higher ammonia or elevated temperatures [171]. The competition among these acetate-consuming organisms results in process stability in different operational conditions and performance of process models that gives complete dominance of a given clade [171]. The main function of *Methanosarcina* (i.e., an aceticlastic methanogen that potentially acts as hydrogenotrophic electron acceptor) is still unclear [172]. In addition, the bacterial community interaction with the acetate-removing community needs to be investigated in future research. Some genetic-scale model (CLM) has been developed to investigate *Methanosarcina* and several key hydrogenotrophic methanogens due to their importance in methane cycling, while *Methanosaeta* has received less attention [157].

Unlike CLM approaches, BPM literature has extensively analyzed sulfate reduction processes where the sulfur-reducing bacteria competes with methanogenesis to harvest electrons and generate hydrogen sulfide as the final electron sink (instead of methane) [173]. Even though some models have been developed in this field, the most challenging aspect of including sulfur reduction is introducing physical chemistry and linking them to iron/phosphorous cycles [153]. Direct electron transfer has been inferred in AD based on CLM analysis. In contrast, BPM-based approaches scarcely distinguish direct versus mediated electron transfer [153,174].

There is a considerable gap between CLM and BPM for mixed culture anaerobic metabolic modelling limiting their application in full-scale AD plants. Even in acetogenesis and methanogenesis where both models are mature in terms of approach and value obtained. The BPMs do not consider the genetic restrictions. This is particularly important when it comes to physical interactions between syntrophic organisms or amino acid transfer. CLMs lack mass transfer and process-related principles such as advection, diffusion and migration. Both BPMs and CLMs are limited by scale and community complexity. This suggest that the best model to describe and predict AD can be a mixed CLM and BPM approach, where the information from microbial community analysis forms rules for BPM and translate BPM principles (e.g., suffusion and ion chemistry including migration) to be used in CLM [153].

### 7.3. Examples of Hybrid Cellular-Level Modeling/Biochemical Process Modeling (CLM/BPM) for Enhanced Predictivity of AD Models

Ramirez et al. (2009) started to include the species diversity within a functional group in the ADM1 model. In this way, they could estimate which organism can take over a biochemical reaction in different operational conditions leading a robust AD process; however, more effort is required to include microbial diversity in ADM1 [175]. In a recent study, intracellular microbial activity data from meta-genomic and meta-transcriptomic analysis was linked with ADM1 in order to investigate the model performance on predicting biogas production from lignocellulosic materials. In addition, the flux-balance analysis of the methanogens actively was continuously updated in ADM1. As a result, this hybrid model could provide detailed information regarding the activity pathways in anaerobic digestion compared to the original ADM1 and could predict the intracellular activity of microbial species that are compatible with experimental data obtained from meta-genomic and meta-transcriptomic analysis [176].

Determined and mapped proteins from meta-proteomic analysis of AD samples not only support the assumptions used in the ADM1 but also revealed some indications of new pathways for biogas production including syntrophic acetate oxidation pathway and other interspecies microbial interactions [94]. Consequently, meta-proteomics can help to identify the changes in functional level and can provide data on the metabolic activity of individual groups. Linking this information with a BPM (e.g., ADM1) may enhance the accuracy of the model and indicate the active pathways for biogas production [147,177].

## 8. Future Perspectives

The main goal for community analysis in AD is to develop a stable and robust process. Therefore, an effort has been made to correlate the structure of microbial community with the process-controlling factors. These correlations cannot directly determine the drivers or barriers of AD; however, they can direct the future research toward developing reasonable process control tools. An extensive number of scientific studies could demonstrate the response of microbial communities against the operation conditions including ammonia concentration, temperature and pH. However, further research needs to be conducted to identify the interaction between different species within the system. This needs a comprehensive database of genetic information of bacteria and archaea that are active in AD.

Application of meta-omic techniques in AD depends on the development of the methods and how quick these methods can identify different aspects of the AD. Meta-proteomics seems to be a promising technique that can be used in AD process; however, research is required to develop an AD biomarker database for various operational failures.

Currently developed AD models consider the microbial community to be one independent population and the models lack information regarding the interaction between different organisms. Even though some researchers could address this weakness, this is a crucial step to develop a general model for AD that simultaneously considers cellular-level activities and biochemical process-level phenomena.

## 9. Conclusions

AD involves diverse communities with high complexity in terms of functional interactions among the individuals or groups of organisms. A joint method including meta-omics, virtualization techniques and chemical analyses may provide a powerful tool for gaining highly valuable information from AD. Such a comprehensive method can provide a multi-disciplinary tool allowing the identification of different species, recognition of their role in the process, distinguishing their function and developing a stable AD process.

The 16S rRNA-based methods have the possibility of integration to an AD process in which the substrate residence time is higher than 2 h (approximate time for real-time sequence amplicon production). This can be a strong tool to correlate variations made by different factors in AD (e.g., feed type, inhibition and operational condition), to the community composition in order to recognize the main contributors in biogas production. Metagenomics expresses the potential of each functional group and can help us to understand the main differences between two similar community compositions. In AD, the main application of metagenomics is to identify the major contributors in biogas production linked to the operational condition; however, the integration of metagenomics in AD is not yet possible due to its slow process rate.

Meta-transcriptomics are suitable tools to determine low-abundance microbes and their contribution to system stability. It can also reveal the multi-functional microbes in the process. Meta-transcriptomes are able to track fast changes in AD, such as variations that happen due to rapid adaptation of the culture. This means that the results of meta-transcriptomics linked to the operational conditions can help to establish an efficient anaerobic digester. At the cellular level, meta-proteomics are powerful techniques to determine the variations in proteins and enzyme levels. Some of the enzymes that can be identified with meta-proteomics can serve as biomarkers to predict future failures within the biological system. In comparison with meta-proteomics, metabolomics can provide detailed snapshots from different pathways. Metabolomics has a high potential to be integrated in AD as a predictive tool in order to enhance the biogas production and avoid process failure; however, the lack of an AD biomarker database has limited the application of meta-metabolome in AD.

## Figures and Tables

**Table 1:** Syntrophic acetate oxidation linked to hydrogen conversion to methane.

Reaction Name	Reaction	∆G° (kJ/mol)
Syntrophic Acetate Oxidation	CH_3_COO^−^ + 4H_2_O → 2HCO_3_^−^ + 4H_2_ + H^+^	+104.6
Hydrogen to Methane	4H_2_ + HCO_3_^−^ + H^+^ → CH_4_ + 3H_2_O	−135.6
Overall Reaction	CH_3_COO^−^ + H_2_O → CH_4_ + HCO_3_^−^	−31

**Table 2:** Some of the biochemical and molecular methods for determination of microbial diversity [47]. Different platforms and sequencing technologies have been listed in detail by Kulski 2015 [52].

Molecular Biotechniques	Biochemical Techniques
Mole percentage guanine-cytosine Nucleic acid hybridization DNA reassociation, restriction fragment length polymorphism (RFLP) Terminal restriction fragment length polymorphism (T-RFLP) Ribosomal intergenic spacer analysis (RISA) Automated ribosomal intergenic spacer analysis (ARISA) Amplified ribosomal DNA restriction analysis (ARDRA) DNA microarrays Polymerase chain reaction (PCR)	Plate counts Sole-carbon-source utilization (SCSU) Phospholipid fatty acid (PLFA) analysis
